# Psychometric properties and relations with coping and family strain of the Health Services and Caregiver Experience questionnaire (HSCE): an outcome measure of informal caregivers’ experience for inpatient care in Italy

**DOI:** 10.1186/s12913-017-2317-x

**Published:** 2017-07-17

**Authors:** Anna Coluccia, Fabio Ferretti, Andrea Fagiolini, Andrea Pozza

**Affiliations:** 1 Department of Medical Sciences, Surgery and Neurosciences, Santa Maria alle Scotte University Hospital, Viale Bracci 16, 53100 Siena, Italy; 2 Department of Molecular Medicine, Santa Maria alle Scotte University Hospital, Viale Bracci 16, 53100 Siena, Italy

**Keywords:** Caregiver, Experience, Questionnaire, Inpatient care, Health services, Psychometric properties

## Abstract

**Background:**

In the last decade, the number of patients supported by informal caregivers has substantially increased. In the Italian healthcare context, informal caregivers’ experience of care is a new under-recognized construct, and no assessment tool is available. Measuring caregivers’ experience is important since in Italy the relationship between doctors and patients/relatives is still considered asymmetrical. The current study presented development and initial psychometric properties of the Health Services and Caregiver Experience questionnaire (HSCE), a self-report tool of caregivers’ global experience for inpatient clinical care, including factor structure, reliability and its relations with measures of coping strategies and family strain.

**Methods:**

The HSCE was administered to a total of 503 informal caregivers of inpatients admitted at an Italian University Hospital (mean age = 48.08 years, SD = 14.82, females = 61.40%). Family Strain Questionnaire-Short Form (FSQ-SF) and Coping Orientations to Problems Experience-New Italian Version (COPE-NVI) were administered to a subgroup of participants. First-grade relatives were 73.10%, whereas 13.20% were second-grade relatives and 13.70% were home-watch caregivers.

**Results:**

Exploratory and confirmatory factor analyses showed a structure with a single factor, which explained 64.80% of the total variance. All the items had salient loadings. In the two subsamples, HSCE had excellent internal consistency (Cronbach’s alpha = 0.95–0.97). Positive moderate correlations were found between HSCE and FSQ-SF scores (*r* = 0.45, *p* < .05), between HSCE and COPE-NVI scale scores, including COPE-NVI positive attitude and COPE-NVI problem solving scores (rs’ range = 0.51–0.57, *p* < .05). Moreover, a positive large correlation between HSCE and COPE-NVI social support scores emerged (*r* = 0.72, *p* < .05). Correlations were not significant between HSCE scores and COPE-NVI turning to religion and avoidance strategies.

**Conclusions:**

The HSCE resulted to have good psychometric properties. Better caregivers’ experience correlated with stronger family strain but also with better problem solving and social support. The study expanded knowledge on caregiver’s experience in Italy and indicated that HSCE is a valid and reliable tool to measure this under-recognized construct in Italy.

## Background

Informal caregivers are family members or natural persons who aid the daily care of a disabled individual by contributing in caretaking responsibilities, even if they do not necessarily live with the frail person in the same house [1]. Recent years have seen awareness of the central importance of informal caregivers in the management of the continuity of care for inpatients with a wide variety of diseases. Caregivers may have expectations of care for their relatives, which are based on their values and experience of the care pathway, interpretations of symptoms, socio-cultural level, and ethnic membership [2]. Improvement of inpatient clinical care requires assessment of key dimensions of health care quality. The U.S. Institute of Medicine included among the key dimensions of healthcare quality concepts of safety, effectiveness, timeliness, patient-centeredness, efficiency, and equity [3]. However, it can be believed that a patient-centered care includes also attention on caregivers’ experience of care, particularly family members. Healthcare professionals not always involve informal caregivers in planning the inpatient’s discharge from inpatient care or ask them how they are coping with caregiving [4]. Research has demonstrated that considering also caregivers’ experience of care might have some advantages from a policy-making point of view. Indeed, higher caregivers’ experience of care is associated to higher family inclusion in the treatment/care decisions, better management of the patient when at home and better quality of life among patients and also caregivers’ themselves [5, 6]. Some research has been conducted investigating the construct of caregivers’ experience. Using Grounded Theory approach, Attree [7] conducted a study on relatives of patients with acute medical problems, reporting that quality of care was described as personalized, patient-centered, related to needs, and characterized by involvement and compassionate behaviours.

Some research studied tools of measurement of caregivers’ experience. Most of the researches focused on the development of tools measuring satisfaction of caregivers of young people with mental health problems [8, 9], caregivers of patients with neurological diseases or in palliative care contexts, such as Dementia [10], and caregivers of older adults in geriatric care settings [11].

Despite the growing interest, to our knowledge, there is a lack of tools measuring experience as a global construct for inpatient settings, which could be used with caregivers of inpatients suffering from different diseases. A tool measuring global experience might help identify similar processes across caregivers of different types of inpatients. Accounting for the experience of caregivers in inpatient settings may also be relevant since the role of caregivers is crucial when the inpatient is dismissed from hospital.

Different from other European countries, the role of caregivers in the Italian healthcare pathway is under-recognized [12]. In the last decade, the percentage of patients supported by informal caregivers has increased from 45% to about 56% [12]. This appears important since in the Italian healthcare context, the relationship between doctors and patients/relatives is still considered asymmetrical, with the latter being viewed as passive recipients of medical instructions [13]. In the Italian healthcare system, the family or informal caregivers of inpatients admitted at hospital wards are limited to a visitor role and are only allowed to see and speak with the inpatient during often restrictive visiting hours. Therefore, informal caregivers’ experience of care is receiving growing attention, but in Italy it is still an under-recognized construct, and no assessment tool is available. Starting from these points, the objective of the current study was to present the development and initial psychometric properties of an outcome measure of caregivers’ global experience for inpatient clinical care, the Health Services and Caregiver Experience (HSCE) including factor structure, and reliability. In addition, its relations with measures of coping strategies and family strain were investigated.

## Methods

### Participants

The HSCE was administered to a total of 503 informal adult caregivers of inpatients admitted at an Italian University Hospital. Mean age was 48.08 years (*SD* = 14.82, range = 18–86). The sample was composed by 61.40% of female participants. First-grade relatives were 73.10%, whereas 13.20% were second-grade relatives and 13.70% were home-watch caregivers. First-degree relatives can be defined as close blood relatives, which include the individual’s parents, full siblings, or children. Second-degree relatives represent blood relatives, which include the individual’s grandparents, grandchildren, aunts, uncles, nephews, nieces or half-siblings. In the Italian healthcare context, home-watch caregivers represent trusted home care providers, specialized in elder care and care for chronic diseases, such as dementia that elder people face daily. Thirty-one percent of the participants were caregivers of inpatients admitted at Internal Medicine care units, 54% were caregivers of inpatients admitted at Gynaecology care units, and 15% of Surgery care units. Gynaecology units consisted of wards, where women who needed to stay in hospital after surgery and emergency admissions for gynaecological diseases or complications related to pregnancy. Data were collected from September 2011 to November 2013. All the participants completed the tool individually or in small groups in the facilities. Eligible participants were caregivers who were attending the inpatient wards of the University hospital. The sample consisted of a convenience sample. Participants were approached in the waiting rooms of the inpatient wards, and were provided with a brief oral and written explanation of the study aims and rationale. Those individuals who were interested in participating, were recruited for the study. When the participants completed the measures in small groups, each of the participants completed her/his questionnaire; thus, each questionnaire pertained to one person and there was not group discussion. Also in the group situation, each participant received a description of the study individually.

### Development of HSCE

The tool was developed by a research staff composed by researchers and health care professionals with experience in the field of inpatients’ and caregivers’ experience, consisting of a sociologist (AC), a senior statistician and methodologist (FF), a psychologist (AP) and a psychiatrist (AF). The HSCE is as an outcome measure of global experience of informal caregivers for inpatient clinical care. The questionnaire was created and then administered to participants in the study in Italian language. It was developed with the aim to cover the aspects related to informal caregivers’ global experience, which were found in the international and the Italian literature as the most relevant ones to inpatients’ caregivers irrespective of the inpatients’ disease and the phase of treatment [12, 13].

The tool is part of a battery of self-report tools created for the assessment of experience of care pathways, including the Health Service & Inpatient Experience questionnaire (HS&PE; [14]) and the Health Services Outpatient Experience (HSOPE; [15]). The questionnaire consists of three sections. The first section comprises ten statements representing experience of a variety of aspects of inpatient care (eg, feeling informed regarding modalities of the relative’s stay at hospital, feeling informed on the visits organizations, feeling provided with clear information when asking questions, feeling prepared to cope with discharge). Question responses are in a 5-point Likert scale self-report format (“Never” =1, “Always” =5). In addition, this section includes also a rating scale with a ten-point response format (“Very dissatisfied” =1, “Very satisfied” =10), aimed to measure overall satisfaction of care. The second section is based on five questions with a closed-response format on demographics of the caregiver (sex, age, and residence, grade of relationship, number of hours spent in hospital). The third section asks suggestions for the improvement of care in terms of caregiver needs.

An electronic search of the international literature was conducted focusing on the caregiver’s experience construct. During staff meetings, the contents of some questionnaires used in previous studies in English-speaking countries were reviewed, including provision of information on the visits and treatment course, humanization of care, caregivers’ involvement in decision making and management of the relative, and overall satisfaction [7, 12–20]. The model of the HSCE was based on defining caregivers’ experience as an unidimensional construct [20, 21]. Subsequently, the staff produced a preliminary list of items and created a pilot version of the HSCE. This version of the tool was piloted by cognitive interviews with caregivers from the University Hospital facilities, where the participants completed the measure, then were asked to provide feedback on the relevance and comprehensibility of the items. This version was considered comprehensible by the participants, then it was used to test for its properties in the present study.

An English version of the HSCE was developed (text of the items is reported in Table 1). The translation procedure into English was made according to forward- and backward-translations. The former was conducted by a native Italian-speaking psychologist having very good fluency in English, then checked by another Italian healthcare professional having very good English proficiency. The two translators discussed the forward-translated version during meetings with a third professional, in order to reach consensus. Finally, this version was translated back into Italian by a bilingual professional translator, who was blind to the original Italian version of the HSCE. The back-translated version into Italian was then compared with the original Italian version, and discussed by the all the translators in a meeting, which lead to the final English version, reported in Table 1.

**Table 1:** Factor loadings matrix of the HSCE items (*n* = 247)

	*λ*_*1*_	*h* ^*2*^
Did you feel at ease in dealing with the staff?	,883	,78
Was the staff reliable?	,855	,73
Were you informed by the staff about the outcome of the visit and the course of the health care pathway for your relative?	,854	,73
Where necessary, were you able to find a doctor who was willing to give you the information you needed?	,838	,70
Was the staff timely when you requested help in caring your relative?	,801	,64
Did you receive clear and comprehensible information on the facility organization from the staff (time of relatives’ visits)?	,779	,61
Did the staff respect your privacy needs during the visits with your relative?	,767	,59
Did you feel that your concerns were taken into account by the staff?	,759	,58
Did you feel involved in decision making regarding your relative?	,674	,45
Did the staff respect your privacy needs during the visits with your relative?	,557	,31

*HSCE* Health Services Caregiver Experience questionnaire

### Other measures

A subsample of 30 informal caregivers (78.90% females, mean age = 52.37 years, SD = 14.35, 89.50% first-grade relatives) completed also the Family Strain Questionnaire-Short Form and the Coping Orientation to Problems Experience-New Italian Version. The subsample was a convenience subsample, recruited in the waiting rooms of inpatient wards as the general sample. Participants in the subsample were approached after having received a detailed description of the study aims and rationale, particularly explaining that the aims were to investigate the relation between caregivers’ experience of care, family strain and coping resources in inpatient wards.

### Family strain questionnaire (FSQ-SF)

The Family-Strain Questionnaire–Short Form (FSQ-SF; [21]), a self-report scale for nurses and general practitioners, is designed to assess perceived caregiving-related problems. It includes 30 items with a true-false response format, and is aimed to measure emotional burden, social involvement problems, the need for knowledge of the disease, the quality of family relationships. It showed very good internal consistency (Cronbach’ alpha = 0.88).

### Coping orientations to problems experience-New Italian version (COPE-NVI)

The Coping Orientation for Problem Experiences-New Italian version (COPE-NVI; [22]) is a self-report scale for assessing coping strategies in the Italian context. It aims to measure different types of potential response strategies to stressful events and is based on five scales, including social support, avoidance strategies, positive attitude, problem solving and turning to religion.

### Data analytic plan

In order to evaluate factor structure, data from the total sample were randomly splitted into two subsamples with simple randomization. The first subsample, composed by 247 participants (subsample 1) was used to conduct Exploratory Factor Analysis (EFA). The second subsample, obtained from the remaining 223 participants (subsample 2) was used to perform Confirmatory Factor Analysis (CFA) by structural equations modelling [23]. The distributional properties of the HSCE items were investigated through the inspection of indices of skewness and kurtosis. In order to examine goodness of fit of the model to the data, the following indices recommended by Hu and Bentler [24] were used: the Adjusted Goodness-of-Fit Index (AGFI), the Goodness of Fit Index (GFI), the Bentler-Bonett Normed Fit Index (NFI), the Bollen’s Relative Fit Index (RFI; [25]). For these indices, values close to 1 suggest a good fit. In addition, the Root Mean Square Residual (RMR) was used; for this index, values less than .08 represent acceptable fit, whereas those less than .06 represent good fit. Reliability was investigated as internal consistency by Cronbach’s alpha coefficients on subsamples 1 and 2 separately according to Nunnally and Bernstein [26] (alpha > .70 = acceptable, alpha > .80 = good, alpha > .90 = excellent). Pearson’s bivariate correlations were computed between HSCE, FSQ-SF and COPE-NVI scores. The analyses were performed through SPSS v21.00 and AMOS.

## Results

### Item distributional properties of the HSCE

In subsample 1, three of the 10 items (“Was the staff reliable?”; “Did the staff respect your privacy needs during the visits with your relative?”; “Did you feel at ease in dealing with the staff?”) reported a kurtosis or skewness value out of the range, suggesting that data on these variables had not normal distributions [27]. The examination of the patterns of response frequencies supported this conclusion, as the majority of participants (74% for the first, 76% for the second, 73% for the latter item) endorsed these items as “Always” or “Often”.

One-way ANOVA analyses showed that male caregivers reported significantly higher scores on the HSCE than females (F_(1, 413)_ = 6.70, *p* < .001). First-grade relatives reported significantly more elevated scores on the HSCE than second-grade ones and other types of informal caregivers; second-grade relatives had significantly higher scores than other types of informal caregivers (F_(2, 388)_ = 23.78, *p* < .001).

### Factor structure based on exploratory factor analysis

The assumptions requested for conducting EFA were supported. The value of the Kaiser-Meyer-Olkin index of sampling adequacy (KMO; [28]) was 0.96, indicating that the correlation matrix was suitable for EFA, since the KMO values should be |0.60| or higher [29]. In addition, results showed that the Bartlett’s test of sphericity [30] was significant, indicating that the data matrix was not an identity matrix (*χ* ^2^_(45)_ = 1566.52, *p* < .001).

**Fig. 1:**
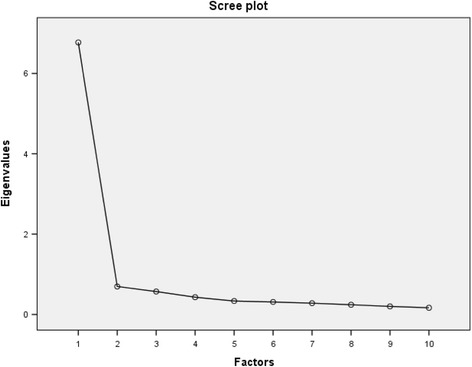
Scree plot of the HSCE items (*n* = 247)

As three of the items did not have a normal distribution, EFA was conducted through the Principal Axis Factoring technique, following recommendations provided by Floyd and Widamann [31]. Three factors had eigenvalues over 1.0: the first one had an eigenvalue equal to 6.48, the second one to 0.70. As recommended by Floyd and Widamann [31], the number of factors to be extracted was identified by the inspection of the Scree plot, which suggested the extraction of one factor. Subsequently, a parallel analysis was performed, where the determination of the 95^th^ percentile for the eigenvalues of items correlation matrix was based on 100 independent random matrices found through the permutation of real data. Findings indicated the presence of a single factor, as the second one of the mean eigenvalues resulting from random data (1.22, percentile = 1.28) was higher than the one found by the real data (0.77). Overall, the single-factor structure explained 64.80% of the total variance. All the items had salient loading values on the single factor, which were higher than |0.30|. Following recommendations provided by Tabachnik and Fidell [29], a value of |0.30| was adopted as the minimum loading. Scree plot and loadings on the single factor are presented in Fig. 1 and Table 1, respectively.

### Factor structure through confirmatory factor analysis

Kurtosis and skewness values for item 1 (“Did you receive clear and comprehensible information on the facility organization from the staff (time of relatives’ visits)?” and 5 (“Did you feel involved in decision making regarding your relative?”) were out of the range comprised between +1 and −1, suggesting that the assumption of multivariate normality was not met. Thus, the estimation method of Unweighted Least Squares was employed. Results confirmed a one-factor solution. However, the RMSEA index was not in the requested range for an acceptable fit (RMSEA = .09). Thus, modification indices were examined. Subsequently, inclusion of covariances between residuals of item 4 (“Were you informed by the staff about the outcome of the visit and the course of the health care pathway for your relative?”) and those of item 5 (“Did you feel involved in decision making regarding your relative?”) improved fit of the model, and the RMSEA value decreased to .07, suggesting acceptable fit. Indices of model fit for the single-factor structure are reported in Table 2.

**Table 2:** Indices of model fit of the single-factor structure of the HSCE (*n* = 223)

Model	*χ* ^2^_(35)_	GFI	RFI	NFI	TLI	IFI	CFI	RMR	RMSEA	*χ* ^2^/gdl
Single factor	104.67*	.91	.95	.96	.96	.97	.97	.043	.09	2.99
Single factor with modification indices between items 4 and 5	78.67*	.93	.97	.96	.98	.98	.98	.038	.07	2.31

*AGFI* Adjusted Goodness-of-Fit Index, *GFI* Goodness of Fit Index, *HSCE* Health Services Caregiver Experience questionnaire, *NFI* Bentler-Bonett Fit Index, *RFI* Bollen’s Relative Fit Index, *RMR* Root Mean Squared Residual

*p < .05

### Reliability

The Cronbach’s alpha estimate was 0.95 in subsample 1 (range of item-total correlations = 0.41–0.79), highlighting excellent internal consistency according to guidelines [26]. All the item-total score correlations were higher than 0.20, as recommended by Nunnally and Bernstein [26]. When each of the items was removed from the scale, Cronbach’s alpha values ranged between 0.93 and 0.94. Also in subsample 2, excellent internal consistency was found, since the Cronbach’s alpha estimate was 0.97 (range of item-total correlations = 0.56–0.86). Alpha values when each of the items was removed ranged from 0.96 to 0.97.

### Correlations between HSCE, family strain and coping strategies

Positive moderate correlations were found between HSCE scores and FSQ-SF scores (Pearson’s *r* = 0.45, *p* < .05), between HSCE scores and COPE-NVI scale scores, including COPE-NVI positive attitude scores and COPE-NVI problem solving scores (Pearson’s rs range = 0.51–0.57, *p* < .05). Moreover, a positive large correlation between HSCE scores and COPE-NVI social support scale scores emerged (Pearson’ *r* = 0.72, *p* < .05). Correlations were not significant between HSCE scores and COPE-NVI turning to religion and avoidance strategies. An overview of Pearson’s correlation coefficients’ values is provided in Table 3.

**Table 3:** Pearson’s bivariate correlations between HSCE, FSQ-SF, COPE-NVI scores (*n* = 30)

	1.HSCE Total	2.FSQ-SF Total	3.COPE-NVI Social support	4.COPE-NVI Avoidance strategies	5.COPE-NVI Positive attitude	6. COPE-NVI Problem solving	7.COPE-NVI Turning to religion
1.	1	,456*	,722**	,346	,509*	,572*	,383
2.		1	,535*	,395	,003	,226	,489*
3.			1	,636**	,597**	,447	,645**
4.				1	,173	,202	,407
5.					1	,614**	,226
6.						1	,398
7.							1

*HSCE* Health Service Caregiver Experience questionnaire, *FSQ-SF* Family Strain Questionnaire-Short Form, *COPE-NVI* Coping Orientation to Problem Experience-New Italian Version

*p < .05

**p < .01

## Discussion

In the last decade, the number of patients supported by informal caregivers has substantially increased. However, in the Italian healthcare context, informal caregivers’ experience of care is under-recognized, and there is a lack of psychometric tools. The current study presented the development and initial psychometric properties of the HSCE, a tool developed as a global measure of experience of caregivers for inpatient care. Psychometric properties were tested on a large sample of informal caregivers of inpatients admitted in surgery, gynaecology and internal medicine units at a University Hospital. Based on previous literature, experience of inpatient care was assessed with regard to several aspects (eg, feeling informed regarding modalities of the inpatient stay and treatment course, feeling informed on the visits organizations, feeling provided with clear information when asking questions, feeling prepared to cope with discharge). The current study seemed to confirm caregivers’ experience as a global construct. EFA supported a single-factor structure, which explained 64.80% of the total variance in all the items, which all had loadings above the chosen cut-off on a single factor. CFA demonstrated that a single-factor structure had good fit. The inclusion of the covariances between the residuals of item 4 (“Were you informed by the staff about the outcome of the visit and the course of the health care pathway for your relative?”) and those of item 5 (“Did you feel involved in decision making regarding your relative?”) showed an improvement of fit in the RMSEA index, which made the fit acceptable. An explanation for this result could be that both items 4 and 5 share some semantic elements which could cover a specific aspect of experience related to being involved in decision making regarding the healthcare pathway of the patient. Thus, this aspect could account for the covariances between residuals of these items.

Reliability as internal consistency resulted excellent for a single-factor solution for both subsample 1 and subsample 2, since Cronbach’s alphas values ranged from 0.95 to 0.97, suggesting that all the items should be included in the scale. With regard to variables associated to caregivers’ experience, results suggested that male caregivers reported better experience of care, and this appeared in line with previous results on caregiving [32]. Overall, this finding could suggest that interventions improving caregivers’ experience should be directed to female caregivers, who could have worse experience of care. As indicated by previous research in Italy [33], female caregivers are more likely to be engaged in tasks related to care provision and long-term management, to suffer from role perceived strains, and experience stronger emotional burden after the inpatient’s discharge than male caregivers, and this could be also associated with higher expectations of being involved in the inpatient home management. Thus, the result found from the current data could be explained by a culturally-based gender division of roles in informal caregiving. In the Italian context, men tend to be less likely to provide informal caregiving and more likely to meet these needs through financial resources.

Interestingly, the current data provided support for a positive correlation between experience of caregivers and family strain, suggesting that those caregivers who reported higher strain tended to have a better experience of inpatient care. These findings appeared in contrast with previous data reporting that caregivers who had less strain had better experience of care [34]. However, the cross-sectional nature of the study design did not provide secure evidence about the causal relation between caregivers’ experience and strain. Future research should investigate more deeply this aspect through controlled designs, in order to verify the direction of this relation. Among coping strategies, coping based on social support was the type of coping most strongly related to caregivers’ experience; problem solving-based coping and positive attitudes were moderately related to experience, whereas turning to religion and avoidance strategies were not related. Overall, these findings expanded previous data, which indicated that problem solving was a coping strategy associated to better experience of care among caregivers [32]. It could be hypothesized that interventions directed to informal caregivers should increase social support, positive attitudes and problem solving, in order to enhance better experience of inpatient care.

Finally, some limitations and future directions for research should be pointed out. Future research is needed in the Italian healthcare context, where patient-centeredness and also caregiver-centeredness of care are understudied. First, the present study did not use a validated measure of caregivers’ experience as a comparator to investigate convergent validity of the HSCE. Another limitation regards the fact that all the participants were recruited in a university setting. Future research should evaluate the usefulness of this questionnaire in other care contexts, such as home care or community settings. A further limitation concerned the use of a convenience sample of caregivers, as it consisted of caregivers who were approached in the waiting rooms of the inpatient wards and were provided with the study aims and rationale. Those individuals who were interested in participating, were then recruited. Future research could overcome this limitation selecting a large random sample of inpatients, whose informal caregivers could be recruited for the study. Another suggestion for future studies could be studying factor invariance across the subgroups of care units, as the current study assessed the factor structure of the questionnaire merging data from three main different wards. Future research should evaluate whether the single-factor structure is supported across the three types of wards. Finally, in the future research, the HSCE could be used as a tool to evaluate the relation between caregivers’ experience of care and the clinical outcomes for the patients.

## Conclusions

In conclusion, the current study expanded previous knowledge on caregivers’ experience, presenting psychometric properties of the HSCE, a measure of global experience, which was tested among informal caregivers of inpatients admitted in surgery, gynaecology and internal medicine units. It demonstrated valid and reliable properties with a single-factor structure and excellent internal consistency. Male caregivers reported significantly better experience than females. Higher experience was associated with stronger family strain, suggesting that caregivers with higher strain had better experience. Adding knowledge on the relation between experience of care and coping strategies, the current study showed that coping based on social support, problem solving and positive attitudes was related to better experience, whereas a relation between turning to religion and avoidance strategies and experience was not found.
